# Sacred Spaces, Healing Places: Therapeutic Landscapes of Spiritual Significance

**DOI:** 10.1007/s10912-014-9318-0

**Published:** 2014-12-13

**Authors:** Geraldine Perriam

**Affiliations:** School of Geographical and Earth Sciences, University of Glasgow, Glasgow, G12 8QQ UK

**Keywords:** Healing, Spirituality, Therapeutic landscapes, Pilgrimage, Christianity

## Abstract

Understandings of the relationship between space, culture and belief are formative in the experience of seeking healing. This paper examines the relationship between place, healing and spirituality in the context of interdisciplinary perspectives (particularly those of the medical humanities) on healing and well-being. The paper examines places of spiritual significance and their relationship to healing in the ‘uncertain’ quest for alleviation or cure, exploring these thematics in the context of the work on the geographies of ‘therapeutic landscapes.’ Through a discussion of fieldwork at two sites in Perthshire, Scotland, a framework is proposed for the investigation of therapeutic sites of spiritual significance, detailing features such as connection, renewal, reproduction, participation, alleviation and expectation. A deeper examination of sites of healing with spiritual significance, it is proposed, has the potential to develop greater understandings of the ways in which people experience illness and well-being.

## Introduction

In the medieval period, pilgrims travelled throughout Scotland to sacred sites such as holy wells, churches, shrines and other sites (Hall 2005). Some of the sites were particularly associated with healing. Such quests for healing or well-being sprang not only from the desire to seek alleviation but also from understandings that the spiritual experience available at these sites would aid recovery. The relationship between place, spirituality and healing continues today as people still seek healing of body, mind and spirit in similar sites. This paper explores these themes in the context of work on the geographies of ‘therapeutic landscapes’, defined briefly as “places that have achieved lasting reputations for providing physical, mental and spiritual healing” (Gesler 1992). The work presented here is informed in particular by that spiritual component and in addition speaks to the under-appreciated role of reputation and belief in the production of such spaces and outcomes.

Perspectives on ‘wholeness’, healing and well-being range across a number of disciplines such as human geography (Atkinson, Fuller and Painter 2012), medical anthropology (Morrison, Tay, and Diener 2011; Storck, Csordas, and Strauss 2000) and social policy analysis (Aldridge 2000). The medical humanities, however, uniquely allow for interdisciplinary exploration of the human quest for healing and ‘wholeness’ beyond technical and physical interventions, drawing on other disciplinary investigations. In the context of such interdisciplinary perspectives on healing and well-being, this paper examines the relationship between place, healing and spirituality. In particular, the paper explores places of spiritual significance and their relationship to healing in the ‘uncertain’ quest for alleviation or cure. Healing can be understood as a quest for ‘wholeness’, which in turn can imply a fragmented self/body that can be ‘put together again’, with disparate elements somehow reformed to constitute a representation of (in this case the ‘healthy’, whole body) an entity that had a previous existence. ‘Wholeness’ as discussed in this paper is defined as an integration of body, mind and spirit rather than a reconstitution of a former (illusory) entity (Wight 2012). Healing is not necessarily cure. In this paper, healing is understood as alleviation, a possible reduction in the severity of symptoms, an improvement in the quality of life “despite disease” (Glannon 2004, 72). Places of healing are sites where there is an intention to provide the means of alleviation or improvement, as well as possible cure. Well known therapeutic sites of spiritual significance, such as Lourdes, intend to offer healing through spiritual (sometimes faith-based) embodied actions, retreat and the opportunity for peace and stillness. Historical understandings of such places reveal that a spiritual dimension to the therapeutic properties of place often involve witness and/or interpretations of healing events such as miracles or shared experience of a gradual improvement in health after attendance at these sites. Belief or hope in the possibility of healing at such sites increases as narratives of healing evolve between individuals and groups who use or access such places.

This paper examines two such sites in Perthshire, Scotland. The first is a small town, Killin, that has a centuries old tradition of healing related to the cult of Saint Fillan. The second is a newer, late twentieth century site at the Bield, Blackruthven, developed specifically for retreat, prayer and healing. Both sites are Christian but neither is exclusive to those of the Christian faith. In order to explore the spiritual dimension of these sites, the paper first examines the conceptualisation of spirituality, particularly in relation to healing. With a focus on the concept of the ‘therapeutic landscape’, as developed by geographers in the early 1990s, the paper then outlines some aspects of geographers’ work in the areas of spirituality, therapeutic landscapes, emotion and place, identifying the significance of this work in relation to the two case studies outlined above. Particular emphasis is placed on the work of Gesler (2003). Through reflection on Gesler’s work in relation to the site of Lourdes, I develop a set of perspectives that are useful as an analytical framework for examining the two sites. This is the focus of the main section of the paper in which I explore the history of these sites and their significance as sites of healing in a spiritual context.

## Spirituality and healing places

The spiritual dimension of therapeutic sites involves teasing out understandings of spirituality. To understand the relationship between place, spirituality and healing, unhelpful binaries of wellness/illness, inner and outer lives, body and mind, need to give way to more nuanced interpretations of human experiences of suffering and pain.

In examining therapeutic landscapes of spiritual significance, this paper defines spirituality as person- *and* place-centred, a means of interdependence, mutuality and connection. Dewsbury and Cloke in their discussion of “spiritual landscapes” suggest that spiritual elements offer up “new imaginations of our place in the world and how that world works” and also present “the unknown” to us (2009, 698). Such imaginations are contingent upon connection. The ‘connection’ suggested here refers to a sense in which self, others and the environment are related, including elemental forces and nature. Spiritual experience is not necessarily religious nor is it always faith-based, and what we might call spiritual experiences of place are often perceived as traversing body and land. Spiritual dimensions of human experience range across faith-based systems of belief, ethical and moral beliefs in standards of human behaviour and heightened sense of purpose. This may also include cultural understandings of what constitutes goodness and strong feelings of compassion and love. Spiritual experience can also include momentary levels of heightened awareness of well-being induced by a variety of physical and non-physical actions, from meditation to evangelical, performative acts of prayer. It is possible for a strongly religious person to have a spiritual experience that does not directly involve any faith-based activity. Similarly, those with no faith-based practices or beliefs may be moved by participation in religious activity. In the search for healing, an experience of stillness or extreme peacefulness often has a spiritual dimension, involving a momentary or longer-lasting relief from emotional suffering and/or pain.

Tanyi examines understandings of spirituality through a discussion of nursing practice and the spiritual needs of patients. Her conclusions are that spirituality:

is a personal search for meaning and purpose in life, which may or may not be related to religion. It entails connection to self-chosen religious beliefs, values and practices that give meaning to life, thereby inspiring and motivating individuals to achieve their optimal being. This connection brings faith, hope, peace and empowerment… and the ability to transcend beyond the infirmities of existence. (2002, 506)

Although the above definition is largely based on religious experience, the concept of spirituality as a personal search for meaning and purpose in life is useful in that it connects with ideas about hope and the value of life beyond mere existence. Wight in consideration of Vernon’s (2008) work, believes that well-being is connected to a human need “for deep meaning beyond everyday life … the sense of being part of something bigger, more transcendent, mysterious, but loving which constitutes a spiritual dimension to wellbeing” (2012, 241). His emphasis on well-being as ‘wholeness’ also argues for an integration of body, mind and spirit, seeing each as connected and inseparable.

Those who seek greater meaning in life may look for this by engaging in practices that promote ethical behaviour, compassion-focused volunteering, environmental campaigning and physical labour used to improve the lives of others or the natural environment (Muirhead 2012; Gesler 2003). Such activities, beliefs and values encompass a spiritual and spatial dimension, a desire to move beyond the everyday, to live those values in particular ways that may be of benefit to other humans, animals or the environment. There is a ‘secondary gain’: the benefit to the individual from helping someone or something else, which also engenders feelings of living a less two-dimensional life of everyday existence. None of these practices may be connected at all with religious beliefs, but they have a spiritual dimension. Such experiences as described by Muirhead (2012) in his exploration of embodied and emotional experiences of those engaged in environmental volunteering, lead him to conclude that the relationship between the environment and the physical and mental well-being of volunteers was linked to “spiritual awareness” as a result of embodied and emotional practice in particular places (149). The environmental volunteers experienced spiritual awareness through their physical labour and emotional engagement with particular sites. The environmental ‘pilgrimage’ of the volunteers to particular places can be expressed as a devotion to the site and the work performed there, which, in turn, leads to physical and mental well-being as well as a sense of achievement; an experience that allows for the “new imaginations” of one’s place in the world (Dewsbury and Cloke 2009). This is echoed in a discussion of landscape and a phenomenology of lived experience and “bodily practice” that is performative (Wylie 2007, 186). The performative nature of pilgrimage and the spiritual practice of seeking a sacred space beyond the everyday (Stump 2008, 334) allow for an expression of spiritual practice that gives access to spaces beyond the limits of daily life. Travel to particular sites in a quest for healing can also involve action as a belief or hope in the value of that site. Motivations and lived experience are expressed in multiple ways from spiritual or medical need to the recreational aspects of the journey (Williams 2010). Williams’ study of pilgrimage and pilgrims at the shrine of St. Anne de Beaupre in Canada examines the multiple connections and outcomes for pilgrims to the shrine. Williams identifies the complexity of the material, symbolic and social aspects of the therapeutic experiences of the pilgrims. Shrines, according to Eade and Sallnow (1991) are also capable of “accommodating diverse meanings and practices”, allowing pilgrims to seek healing and/or retreat in a variety of ways (15).

The landscape itself offers retreat from daily routine in similar ways to the experiences of the environmental volunteers described above. Spiritual practice can be expressed in particular ways for particular places, perhaps in ways that differ from the ‘routine’ spiritual practices that occur, for example, with weekly church attendance. Pilgrims are able to focus their energies on the physical, emotional and spiritual demands and the outcomes of their journey as it takes them from their everyday lives to new places connected with healing and/or spiritual significance (Schmidt 2009).

Spiritually significant experiences can occur in a number of ways, then, as identified in the literature and in many different forms and settings. Common threads among spiritual experiences are the recognition of some greater meaning or dimension to life, emotional engagement at some deeper level, even if momentary, a sense of purpose and the connection of all of these elements with well-being, whether physical, emotional or both. That such experiences are often connected to place and indeed, particular places, are of interest to a spatially sensitive medical humanities. That such connection may also be related to an experience of physical or emotional trauma such as illness, bereavement or disruption in some way has led humans to ascribe meaning to particular places for the possibilities they may promise in alleviating these problems: places are significant in the search for healing, hope, retreat and resolution of one form or another (Gesler 1992, 2003).

## Therapeutic landscapes, spirituality and geography

The concept of ‘therapeutic landscapes’ was developed by Gesler (1992) to encompass a broader range of settings, including those written about in fiction (Baer and Gesler 2004). It became a well-established concept for geographers and social science researchers (Williams 1998, 2010; Rose 2012; Foley 2013). Gesler’s early work in particular was concerned with physical locations considered to be beneficial to healing and well-being in health settings (1992). Williams’ (2007) overview of the development of therapeutic landscapes as a concept since Gesler’s first explorations demonstrates a range of research not just into traditional health care settings but also work on emotional geographies and cultural understandings of healing and spirituality. My own research on therapeutic landscapes in relation tofiction has explored the ways in which writers use their settings as therapeutic landscapes that offer escape and/or a means of ‘writing out’ emotional difficulties (Perriam 2010). In these circumstances, the fictional therapeutic landscapes are a conduit for writers, a quest for healing that is served by developing imagined worlds. Baer and Gesler (2004) offer a similar reading of *The Catcher in the Rye* in their investigation into the escape for the central character into imaginary landscapes that he imagines as ‘real’ in order to evade his precarious mental health and a difficult situation. For some writers, their fictional therapeutic landscapes are retreats from emotional and psychological difficulty, and their landscapes, while therapeutic, may also be landscapes of turmoil. They may be imaginary landscapes, but they are no less complicated than actual, physical landscapes (Philo 2002). The writer’s quest for healing in many circumstances bears some resemblance to the concept of transformative pilgrimage, with both the writing of fiction and the pilgrim’s steps having similar trajectories of journey. The nature of the therapeutic landscapes may vary, but the performative nature of the quest in each case has some similarity. Just as the pilgrim sets out on a journey, reaching particular stages (leaving behind, setting out and return) writers, too, journey through differing stages, performing different acts. Writers leave behind and set out as they commence their work, journeying and arriving at their new world, their created place and returning once the work is complete (Schmidt 2009).

There is, then, a range of ways we can understand therapeutic landscapes. It is spirituality, however, that Williams (2007) identifies as the most challenging aspect of therapeutic landscapes, owing to the subjective nature of spirituality and the need for critical reflection on it. Williams’ work identifies the elusive quality of the spiritual dimension of therapeutic landscapes. As the concept of the therapeutic landscape has extended beyond mainstream health settings such as hospitals so has the concept of what constitutes healing. Forms of healing that are labelled ‘alternative’ often involve traditional practice and are embedded in the historical evolution of culture at particular sites. Those associated with the cult of sainthood offer healing attributed to particular historical figures. Others offer respite and retreat into stillness in the hope of allowing healing of body, mind and/or spirit (Conradson 2007). Healing can also be sought in alleviation, not only of bodily symptoms but also of grief. Memorials and shrines offer comfort and sites of meaning-making to the bereaved. (Miller and Crabtree 2005; Maddrell 2009). The process of questing is embedded in healing places whether spiritually-centred or not, but they are, as Miller and Crabtree assert, learning landscapes, where “hope flourishes over time” (Kong 2004). All of these examples are ones bound up with spirituality of some form or another.

Sacredness, spirituality, faith, religion and belief are all terms that jostle about in work by geographers. It is hard to separate these out when puzzling through the significance of such concepts in relation to healing and place. Spiritual beliefs need not necessarily have formation under the umbrella of organised religion, but they may do so or may have their derivation from personal experience of faith-based communal worship in a number of ways, from attendance at various communal rituals to a loose, childhood connection with church-going. It is impossible to separate the strands of individual belief and worship from communal practice and institutionalised ritual. As Maddrell (2009) explains in her research on belief and bereavement, there is a “blending of sacred and secular in place meaning, where the politics and poetics reflect religion, community and identity” (233). Kong’s (2004) work on the intersection of the sacred and the secular also argues for the complexity of places regarded as sacred. At therapeutic sites of spiritual significance, even for those who have no particular belief or adherence to a faith tradition, they may see spiritual benefit from the site or from the activities enacted there.

Meaning-making and place in the form of religion, particularly organised religion, is another aspect of belief that has led to renewed investigation by geographers. Brace, Bailey, and Harvey (2006) point out that the neglect of religion as an axis of geographical enquiry leaves geography in a “weak position” when engaging in current debates (29). And while the study of religion is not necessarily the study of spirituality or of the sacred or the divine, it is nonetheless an important aspect of geographical enquiry and the same could be said of spirituality. Much of the work by geographers on belief and spirituality has led to a ‘spiritual turn’ in the relationship between geography and the humanities. While these concepts (belief, spirituality, religion) are *not* interchangeable, they *are* relational. Kong (2004) identifies the interconnectedness of what she calls the “cross-cutting relationship” of the “poetics and politics” of “sacred place” (367). For Kong, sacred places are also contested spaces, where it is the making of a particular place into a sacred space that is worth investigation.^1^ In her work on the changing geographies of religion, Kong (2010) cautions that researchers must not take for granted “the meaning of religion and the sacred” (770). Assumptions about spirituality and particular faiths may not stand up to the evidence to be found at particular sites or practices. As Brace, Bailey, and Harvey (2006) explain, the taken-for-grantedness about the interplay of power and religion as tools of the state is often in evidence without deeper exploration of the complexities of individual and collective spirituality and ideas about sacred space. Massey (2005) argues for configuring space as “always in process” and for the acknowledgement of the multiplicities, “fractures” and “dynamism” of space (11). Sacred spaces are beyond simple dualisms or narrow definitions of religious sites, and this is one aspect of Foley’s (2010) work on holy wells in Ireland that is particularly exciting: the “interplay of individual and cultural meanings” (175). It is the locational and relational interplay of such meanings that contribute to the significance of a site. Such webs of meaning allow for complex and nuanced responses to the landscapes.

Dewsbury and Cloke (2009) refer to “spiritual landscapes”, which, for the authors, means moving beyond religion to “open out spaces that can be inhabited, or dwelt in different registers” (696), echoing Foley’s (2010) reference to the interplay of meanings for particular sites. Spiritual landscapes, as Dewsbury and Cloke identify them, are slightly different from sacred spaces. For the authors, spiritual landscapes have the capacity to develop a sense of community while not necessarily being sites regarded as sacred.

The use of a term such as spiritual landscapes in this paper is envisaged as a broad vista in which unbelief and perplexity share space with past and present practices, with wider manifestations of what it means to experience spirituality. Sacred spaces are *part of* the spiritual landscape. The spiritual landscape of a monastery, for example, or a place of retreat, such as the Bield, introduced below, may be religious in derivation and even sacred but the response of visitors may vary. While they might relish the stillness and peace, visitors may not be particularly touched by the sacred in these settings. Stillness, as Conradson (2007) points out, is relational and for some people who visit monasteries for example, their quest for stillness may “simply reflect an appreciation of stillness in more secular terms” (34).

What follows is an attempt to provide a framework for perspectives on therapeutic landscapes of spiritual significance. Using participant observation over two to three days in summer 2010, two sites in Perthshire were visited. The two sites are introduced and described with the subsequent section a summary discussion of key emergent themes from both.

## Therapeutic landscapes of spiritual significance in Scotland

The sites explored for this paper are both in Perthshire. The first site, Killin, has a long history of healing associated with Saint Fillan and the second site, the Bield, is a contemporary venue, an older house and environs recently developed as a site of retreat. Both sites attract visitors and guests and both are associated with both spirituality and with healing.

### Site 1: Saint Fillan and Killin

There were several saints known as Fillan (little wolf in Scots Gaelic), but the Saint Fillan who is associated with Killin is Saint Fillan of Strathfillan in Perthshire, Scotland. Saint Fillan is said to be the son of Saint Kentigerna and is often credited with bringing the two ethnic groups of the Picts and the Scots together through religious conversion. Perthshire abounds with places associated with Fillan (Taylor 2001) who is thought to have founded a monastery in Strathfillan close to Kirkton Farm near the falls of Glen Dochart. This is also where Saint Fillan’s holy pool is located, seen as being beneficial for those with mental health problems.

**Fig. 1:**
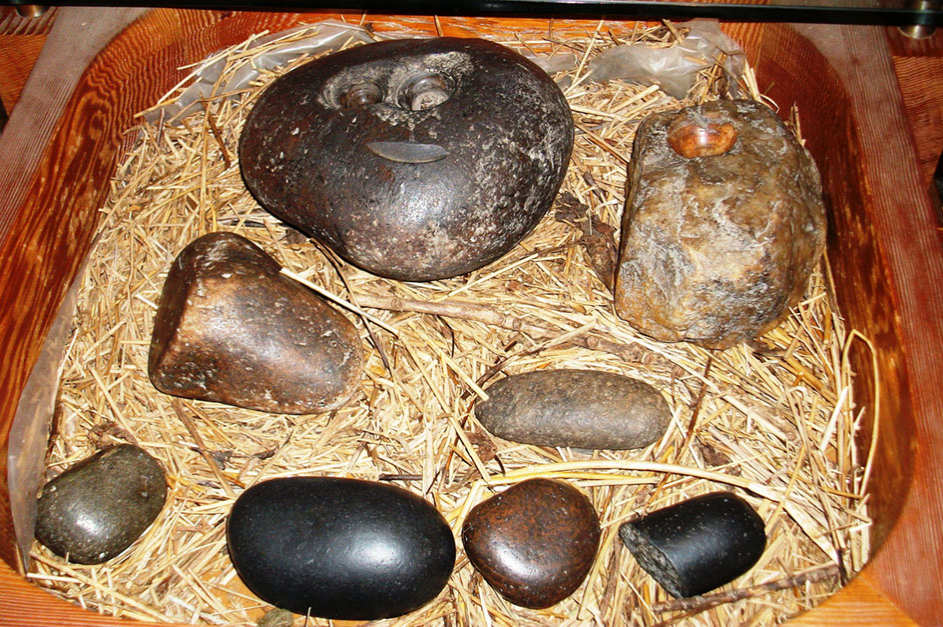
The healing stones of Saint Fillan, Breadalbane Mill, Killin (Source: Author)

The site at Killin that is of particular focus in this research is the village of Killin. In Killin there is an ancient mill that houses the healing stones of Saint Fillan [see Fig. 1] where they have been for centuries. The mill is now the Breadalbane folklore centre, housing a display upstairs devoted to Saint Fillan and his life. The healing stones, located on the ground floor, are available for use upon request and comprise eight, river washed stones laid on a bed of river wrack and straw. Traditionally the stones were selected by those seeking healing to match the particular part of the body that was ailing. Although use varies, staff at the centre have indicated that the stones are usually passed three times one way around the affected area and three times the other way. People continue to visit the centre to use the stones and come from abroad as well as from the village itself.

One of the features of healing associated with Saint Fillan is the distinctiveness of its location. The site of Killin is for bodily ailments, and the Holy Pool near Glen Dochart is associated with illnesses that are psychological in nature.^2^ There are two distinct and separate sites, both with rituals associated with them that are particular to each site. Although both places form part of the cult of Saint Fillan in the area, their therapeutic value remains distinctive. Those who seek healing at either place need not necessarily profess Christian faith. Each person seeking healing is allowed to find his/her own way of performing in these spaces, either using the traditional methods or by making an individual path through the healing process. The components of health performance are shaped here, as Foley (2010) has asserted in his work: by bodies, cultures, spaces and economics. While the site is small and the folklore centre struggles to stay open each year, the economics of Killin are intertwined with the mill and the presence of the healing stones. Additionally, there is renewal enacted through repeated visits and a continuity of practice that is played out in the ritual use of the stones and in the particular rituals in place for caring for the stones. There is also the public element of healing as enacted in these spaces devoted to Saint Fillan for each location is open to the public. The mill site is embedded in the everyday. Staff routinely use the stones, as do local residents.

The geography of the area has played an important role in the continuity of the cult of Saint Fillan and the healing associated with his presence in the area. As Taylor (2001) points out, the medieval parish of Killin was on the main thoroughfare between the Western Highlands and the central belt. Travel through the area and endorsement from King Robert I ensured the continuation in seeking healing from the healing stones of Saint Fillan and the holy pool, as well as diffusing information about their presence in the area.

As a therapeutic landscape of spiritual significance, Killin demonstrates continuity of practice. The association with Saint Fillan and his ability as a healer as well as the cult of Saint Fillan in the wider area of Perthshire is embedded not only in the form of the healing stones and the cultural display at Breadalbane mill but also in the bodies of those who use the stones and those who visit the site.

The site’s association with Saint Fillan and his renown as a healer has developed a practice that is known both locally and in the wider world. The openness with which the healing stones are displayed and allowed for use by visitors and local inhabitants is important to the experiences of healing at the mill. Saint Fillan’s reputation as a regional saint and a continuing force for healing is embedded in both place and the embodied practice of using the stones. As the tourist literature at the Mill suggests, people have been visiting the site for centuries in order to use the stones. Staff at the centre claim that they themselves use the stones regularly with frequent success, while they also recorded regular local visitors calling in to use the stones and reporting back a therapeutic outcome.

### Site 2: The Bield at Blackruthven

The Bield at Blackruthven is a much more modern site, having been open as a place of retreat and healing for little more than ten years. Although Christian in its ethos and practice, the Bield is open to all visitors. Bield is a Scots word meaning shelter, refuge or place of protection. The interpretation of the community that operates at the Bield is that the it can also “express such activities as: to nurture and succour, to embolden, to encourage, each of these meanings suggests a facet of life at the Bield at Blackruthven to which [guests] are warmly welcomed” (The Bield 2012).

The stated ethos of the community is “a centre for Christian spirituality, retreat and healing in the widest sense of the word” (The Bield 2012). It also values nurturing of both soul and body, encouraging learning and reflection. There is a range of options for guests who may visit for a day or for longer periods. There is no expectation that all visitors participate in Christian worship on the site, but it is open to all with two prayer services each morning and evening. There are also courses that run on a regular basis on many aspects of spirituality, healing and personal development. There are resources such as prayer rooms and books are on offer at the site, as well as specific and individual spiritual direction.

For healing there is art therapy organised by a qualified art therapist, prayer, counselling and psychotherapy of various types, gardening, massage and aromatherapy. Attached to the Bield is a small, organic smallholding that “seeks, through therapeutic work, to create a more inclusive community. It… aims to build up self-confidence of people who normally endure long term unemployment and who may feel undervalued” (The Bield 2012). The community includes people with learning disabilities, mental health problems and social difficulties. The smallholding runs an organic box scheme to the local community.

The Bield also has its own organic garden and sustainable energy supply. It endorses a simple, community approach to living together, similar to that practised by the Iona Community. Residents make and change their own beds and clean up after meals which are simple and nutritious in the Iona tradition. This is not a luxury retreat, nor is it ascetic. The aim is to balance the needs of the individual with those of the community and its focus on spirituality and healing.

**Fig. 2:**
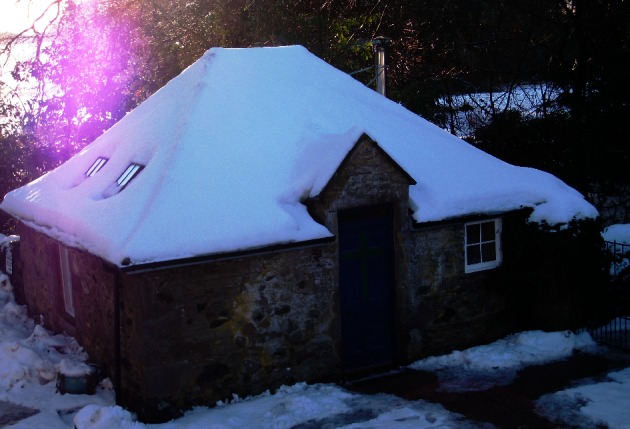
The chapel at the Bield, a former blacksmith’s forge, where worship and prayer are conducted each day (Source: Author)

The site of worship is an old blacksmith’s forge with simple wooden benches and a wooden floor [See Fig. 2]. The bedrooms for guests are simple as are the eating, recreational and spiritual areas. This place is neither remote nor in the heart of a village. The site is quiet and away from traffic, but it is not in any sense removed from the life around it, which is a farming community, only a few minutes’ drive from Perth. Visitors come from diverse backgrounds and anyone in need is accommodated at a moment’s notice. Those coming to the Bield may seek stillness (Conradson 2007), retreat from daily existence, spiritual direction and enlightenment. In some cases they may be looking for specific attention to their emotional well-being through counselling and/or prayer or other forms of therapy. This is a site of renewal, a place where people can come for restoration or repair through attendance at a course, through prayer, silent contemplation or by undertaking spiritual retreat exercises.

The site offers a continuity of Christian worship and prayer. It is a new site, developed to fulfil a spiritual purpose and the holistic aims of the community in providing healing experiences on many levels, an engagement with sustainable practice in conjunction with healing, and Christian worship. There is no border where healing is enacted outwith the spiritual ethos of the site, nor one where spirituality is seen as a separate entity. As a therapeutic landscape of spiritual significance, the Bield is a site where individuals and groups as well as the more permanently based community are invited to enact and embody spiritually significant therapeutic practice. Various practices are enacted daily such as common worship, art therapy, counselling, private prayer and meditation, as well as voluntary work in the garden and grounds. Through work, worship and contemplation, visitors and staff perform acts of spirituality and healing through the course of each day.

## Framing spirituality, healing and place

Having introduced the sites and identified key thematic perspectives within the literature review, I expand here on their significance both as locational and relational perspectives and develop an analytical framework with which critically to understand the two sites under scrutiny. In essence, four broad themes emerge from the sites and these are listed in turn below.

### Connection

Individuals and groups experience connection with a place of healing *or* with others whose presence has similar meaning. The process of connection is complex, involving social, emotional and spiritual engagement with others. Culturally specific beliefs, personal, emotional needs and desires may foster connection that is place-based or expressed as a spatial, even recreational connection (Williams 2010). Pilgrims may experience such connection from a joint motivation or experience of pilgrimage to a site of healing. Communal worship both on the journey and at the site, prayer, sharing of meals and other activity may engender a sense of shared, spiritual purpose. That shared purpose may be centred on the site itself due to the cult of the space or its dedication. In Scotland, the saints Brigid, Columba, Cuthbert, Ninian and Fillan have particular meaning, and certain sites associated with the life and work of the saints will have significance for those who find spiritual connection with them and with other visitors who share their beliefs or experiences. Shared experiences of illness may engender connection with others and with particular sites. That connection may come in the form of the particular healing associated with the site. Specific holy wells and pools have significance for certain disorders such as skin conditions or mental health problems where healing has been historically recorded and observed. Wight’s (2012) position is that connection is entangled with meaning, purpose and understanding, the search for “right” relationship, which is for him, the “essence of spirituality” (15). Connection, in this view, is one of linking the strands of space, self and others in relation to place as a fundamental relational geography.

### Expectation and alleviation

Those who connect with particular sites come with an expectation of healing either due to the historical associations with healing (such as Lourdes) or because of the site’s affiliation with a particular saint and/or healing for certain conditions. Just as one would visit a medical facility in the expectation of healing, so people come to therapeutic landscapes for the same reason. The travellers and local inhabitants who visit Breadalbane Mill do so in the expectation of being healed (or finding some alleviation of symptoms) by the stones of Saint Fillan. This is stated in the tourist literature and by the staff at the Mill. The expectation that healing may be found at particular sites is tied to a desire for alleviation, meaning a temporary respite from a condition or a reduction in pain, not necessarily a permanent cure. A holy well known for its healing properties in the treatment of psoriasis, for example, may be visited for alleviation rather than a cure when the condition is chronic (Gillies 2008). It is not necessarily an expectation of a miraculous cure that may motivate people to visit particular sites of healing but a quest for alleviation, a belief in the power of the site to promote, if not well-being, ‘better-being’, and alleviation for mind, body and spirit may take different forms, despite place-based rituals. At the Bield, several options are available for alleviation from art therapy and Ignatian counselling to the laying on of hands. Motivations for visiting therapeutic landscapes such as shrines and places of retreat are multiple and diverse but the expectation that some benefit may be derived from the visit and from activity there is evident, as the work of Foley (2013, 2010), Williams (2010) and Gesler (2003) demonstrates.

### Participation

Some of the benefit may be from participating with others at the site or, in the case of pilgrimage, sharing the journey (Williams 2010; Morinis 1992). Participation, whether it be in spiritual or physical attempts at healing is performative, an active involvement. It is not a passive surrender of the body to healers but a taking-part in the healing process. Taking part individually or in groups may, in fact, be critical to the healing process – active involvement in determining one’s well-being. Muirhead’s study (2012) of environmental volunteers points to the benefits of active participation in the planned, physical work they undertook. Williams’ (2010) identification of the recreational aspects of visits to therapeutic landscapes indicates that seeking healing may also incorporate participation on a social level. Again, active involvement may have benefits beyond the confines of seeking physical healing in certain contexts.

Participation can, however, be emotionally and spiritually restrictive as well. Church-going and participation in the community may be performed because of expectation, even oppression or repression. Parr, Philo, and Burns (2005) suggest that the romantic and idealised version of the Scottish Highlands (one that has led to an economically successful tourist trade) is counter-constructed by a certain repression, particularly in relation to those with mental health problems. Eade and Sallnow (1991) observe, too, that pilgrims can be restricted by “officials” who “impose their own definitions of the situation”, thus restricting modes of participation (11).

As with externally restrictive practice, individuals can also impose restrictions on themselves. At the Bield, it is possible to participate minimally in the life of the community thereby opting to be ‘on silence’, in other words, not communicating verbally with others, or sharing meals with them but rather, seeking stillness and peace through inner contemplation and prayer (Conradson 2007). The guests who opt to be on silence are still participating in the daily life of the community albeit at some distance from others. By using the facilities - prayer rooms, art room, walking paths, visiting the chapel, working in the garden or simply sitting in a bedroom, praying – guests on silence are still, in a monastic sense, part of the community. The monastic basis of organisation allows for silence, but the daily rhythm of life at the Bield also enables varying forms of participation. Volunteers who work in the gardens participate through their work, often in the monastic tradition of prayer and work, when they also participate in communal worship. The communal worship also invites participation through *lectio divina*: the monastic practice of contemplation and meditation on Christian scripture. This is practised at morning and evening worship at the Bield. Guests and members of the community are invited to reflect on the daily scripture readings either verbally or silently.

Participation in the process of seeking alleviation or healing through the spiritual dimension may take many forms and can operate at many levels. The sense of ‘communitas’ (Turner 1974), “a state of unmediated and egalitarian association between individuals who are temporarily freed of hierarchical secular roles and statuses which they bear on everyday life” (Eade and Sallnow 1991), may not always be evident.^3^ Participation, however, while taking many different forms, is a critical feature of the quest for healing at spiritually significant therapeutic landscapes.

### Renewal and reproduction

People who visit places of healing or therapeutic landscapes often do so in the expectation of a renewal of the healing experience, refreshment from repeated activity such as prayer, bathing or staying for a number of days at a particular site. Renewal, in the Christian tradition, can often take the form of pilgrimage or retreat. Visiting the Bield provides a chance to re-encounter healing or to find alleviation in a spiritual dimension through prayer and other activity, including the physical ‘laying on of hands’ offered for all suffering or illness, both physical and emotional. Renewal of the spirit is also practised through stillness and through activities that allow time for contemplation and quiet, such as working alone in the gardens, using the art room or praying by oneself in the chapel. Renewal is sought by living, for a time, in an extraordinary way, out of the usual routine of one’s daily life. It is, however, daily life for those who live and work at the Bield, “ordinary people”, living in “an extraordinary way” (Norris 2003, 200). Their renewal, in the monastic sense, comes from participating in the daily rituals of prayer and work, as well as offering healing in various forms to guests.

There are similarities with those who work in hospitals and hospices. For patients, these sites are extraordinary, out of the usual life and work patterns, but for staff and the work they do, while it may follow a routine, it is a form of renewal of the healing process. Patients come and go, but staff continue to provide renewal in the form of care and the practice of medicine. Others reproduce and share their experiences in a number of ways: through oral narrative, writing, photography, icons, souvenirs and by digital means such as interactive websites Gesler (2003) notes the “recurring cycle of … witness” among pilgrims to Lourdes (77). The continuity of practice grows through the reproduction of experiences, which are then reproduced by others. Material artefacts such as advertisements and tourist brochures also inform reproduction, as do visitors’ books (Gibson 2012).

At Breadalbane Mill, the use of the healing stones by many and repeatedly in the search for healing or alleviation is part of a thread of continued practice, manifest in the displays, brochures, radio programmes and in the cycle of witness via oral testimony including those who work at the Folklore Centre in the mill. Reproduction of the healing experience can come through ritual where many visitors opt to use the centuries old tradition of passing the healing stones of Saint Fillan three times one way and then in reverse three times. Continued use of the stones and continued evidence of use, via oral and written testimony, allows for the cycle of witness to continue. In the 21st century, reproduction occurs through use of the internet with weblogs, tourism websites and other, interactive sites. Virtual pilgrimages can be made also, allowing those with appropriate technology to reproduce an experience of pilgrimage (albeit in a slightly different manner) through using the internet.

## Conclusion

Therapeutic landscapes of spiritual significance allow for cultural and personal quests for alleviation, connection and renewal. Such sites invite embodied practice of spirituality through various forms of worship, cultural and material activity, many of which may operate in conjunction with, or outside, organised religion. The multiple, relational dimensions of these sites may encourage individual interpretation and quest while accommodating traditional religious practice. They also offer alternatives to those who, for various reasons, find these sites more inviting or approachable than traditional sites of worship such as churches and temples.

Through the application of Gesler’s (2003) work on therapeutic landscapes, I have extended and developed a set of perspectives that can be identified for particular therapeutic landscapes and the spiritual practices enacted at these sites. The two sites profiled in this paper, one of long-standing tradition and one of more recent development, both Christian in origin and ethos, differ in their approach to visitors in the ways in which visitors engage with spiritual and healing practice at the sites. The Bield offers a more holistic experience to guests, whereas the healing stones at Killin are for a specific healing purpose tied to the cult of Saint Fillan. Nonetheless, both contain perspectives of renewal, participation, connection, alleviation, expectation and reproduction. These are demonstrated by the cultural, material and spiritual interpretations of the sites and through the embodied practices of visitors.

Further exploration of these sites and other similar sites in Scotland involves deeper probing of the expectations and experiences of visitors to such sites. Examination of visitors’ books, online commentaries and other modes of response would enable further investigation of these sites and the ways in which people engage in quests for healing through spiritual means. Preliminary observations suggest that place-based forms of spiritual encounters with healing are complex and varied but that the features of these encounters are often experienced by many who travel to such sites. The engagement of mind, body and spirit at these sites is performative but evident in diverse ways, such as ritualised practice, worship (including the culthood of saints), stillness and peace through activity such as gardening or art. Renewal may be sought individually or through group activity and may be recreational, as Williams (2010) points out as much as spiritual. Although both sites are inclusive of those who are not Christian, they are, nonetheless, culturally specific in the forms of worship and/or cult of sainthood they espouse. That both sites are known *beyond* their faith-based ethos indicates that visitors who are not Christian are open to the therapeutic value of the sites in terms of healing.

Spatiality underpins the emotional and spiritual aspects of the quest for healing. Understandings of space and the relationship between space, culture and belief are formative in the experience of seeking healing. How people perceive their place in the world (Morrison, Tay, and Diener 2011), the quality and meaning of their lives, and their experiences of illness and/or healing are dependent on the spatiality of spiritual and emotional encounters. *Where* people seek or experience healing relates to their expectations of particular spaces, their imaginings of how healing may occur there (Storck, Csordas, and Strauss 2000) and the narratives of others, circulating and informing. For some, the simple act of rotating stones about the body may be enough, while for others, praying, participating in worship, silent reflection and the laying on of hands may be deemed more appropriate or more personally and spiritually satisfying. The spatiality of the perspectives of therapeutic sites of spiritual significance extends beyond sites such as those outlined here and is equally applicable to more traditional sites of healing such as hospitals, day surgeries and hospices. In a medical humanities context, the spatial (and particularly geographical) perspectives of the relationship between place, spirituality and healing have the potential to develop further understandings of how people experience and understand illness and well-being.

## Endnotes

^1^ Stump (2008) suggests that sacred space “can be understood as… a religious component of the spatial imaginations of believers that takes different forms in different contexts” (26). Such spaces may not necessarily be religious in derivation but the recognition of the role of “spatial imaginations” is nonetheless important. Rose (2012) emphasises the benefits of “imaginary or metaphoric” representations of landscapes for therapeutic purposes, particularly in the context of psychotherapy (1386).

^2^ Details of the area’s associations with St Fillan were covered in the BBC Radio Scotland Series, ‘On Sacred Ground’, episode 2, 13 January, 2008. Simon Taylor and Gilbert Markus explored the cult of Saint Fillan in Perthshire. Gilbert Markus kindly provided further details in personal communications.

^3^ Eade and Sallnow (1991) demonstrate this through examining critiques of Turner’s model.
